# Preparation Optimization, Rheological Performance and Mechanism of High-Density Polyethylene and Crumb Rubber Composite-Modified Asphalt

**DOI:** 10.3390/ma19183996

**Published:** 2026-09-19

**Authors:** Boyu Hu, Hui Wang, Yijun Tang, Yuehong Huang, Yufeng Tang, Binyang Xu

**Affiliations:** 1 School of Transportation, Changsha University of Science and Technology, Changsha 410114, China; 13574092322@163.com (B.H.); 15806658694@163.com (Y.T.); 15526421451@163.com (Y.H.); tyfdemail@gmail.com (Y.T.); 15273826864@163.com (B.X.); 2 National Engineering Laboratory of Highway Maintenance Technology, Changsha University of Science and Technology, Changsha 410114, China

**Keywords:** composite-modified asphalt, crumb rubber, high-density polyethylene, performance, preparation process, modification mechanism

## Abstract

The application of crumb rubber (CR) and high-density polyethylene (HDPE) in asphalt modification provides an effective approach to improving binder performance while promoting the sustainable utilization of waste materials. However, differences in the physicochemical characteristics of rubber and polymer modifiers make it challenging to achieve a balance among different performance requirements while maintaining structural stability in the composite system. In this study, an HDPE/CR composite-modified asphalt binder was prepared, and the effects of the preparation process and modifier system on the binder performance were investigated. The orthogonal experimental design was employed to determine the preferred preparation conditions, and the properties of the composite-modified asphalt were evaluated through conventional binder tests, rotational viscosity test, dynamic shear rheometer (DSR) test, multiple stress creep recovery (MSCR) test, bending beam rheometer (BBR) test, Fourier transform infrared spectroscopy (FTIR), and scanning electron microscopy (SEM). The results showed that the preferred preparation process improved the overall performance of the HDPE/CR composite-modified asphalt, including high-temperature deformation resistance, low-temperature crack resistance, and thermal storage-induced separation. The incorporation of auxiliary additives enhanced the dispersion and interaction of the CR and HDPE phases and promoted their interfacial interaction, contributing to the formation of a more stable composite structure. FTIR and SEM analyses revealed that the performance improvement was mainly associated with changes in functional groups, interfacial interactions, and modifier distribution within the asphalt binder. These findings provided insights into the preparation process and modification mechanism of HDPE/CR composite-modified asphalt and demonstrate the potential of combining waste rubber and HDPE to develop high-performance and more sustainable asphalt binders.

## 1. Introduction

Asphalt binders are widely used in pavement engineering because of their favorable engineering performance. At the same time, the increasing generation of waste plastics and end-of-life tires has created challenges for sustainable waste management because these polymeric materials are resistant to natural degradation. Their incorporation into asphalt binders provides a promising approach for waste valorization while potentially improving binder performance [1,2,3,4,5,6,7,8,9,10].

Olefin modifiers, including polyethylene (PE), polypropylene (PP), ethylene–vinyl acetate (EVA), and ethylene–propylene–diene monomer (EPDM), have been widely investigated for improving the high-temperature rheological properties and resistance to permanent deformation of asphalt binders [11,12]. Polyolefin modifiers can absorb and swell by taking up the lighter fractions of asphalt, resulting in a two-phase morphology consisting of a polyolefin-rich dispersed phase and an asphalt-rich continuous phase [13]. Various waste polymers, such as low-density polyethylene (LDPE) and ethylene–vinyl acetate (EVA), have been investigated as asphalt modifiers [14,15]. Among these materials, high-density polyethylene (HDPE) has attracted attention because of its wide availability, good thermal resistance, and chemical stability [16]. Previous studies have shown that HDPE-modified asphalt exhibits improved resistance to permanent deformation, high-temperature stability, and aging resistance [17,18]. However, its relatively poor low-temperature ductility and limited storage stability at elevated temperatures remain important limitations [19,20].

Crumb rubber (CR), mainly derived from waste tires, can provide complementary benefits to HDPE by improving the low-temperature flexibility and toughness of asphalt binders while also enhancing their high-temperature shear resistance [21]. However, the incorporation of CR generally increases binder viscosity, and its dispersion and swelling behavior depend strongly on CR content, rubber characteristics, and asphalt composition [22]. In addition, the rheological and morphological characteristics of CR-modified asphalt are strongly affected by processing and digestion conditions, and prolonged hot storage or excessive digestion does not necessarily improve binder performance [23]. Combining HDPE and CR therefore offers potential for balancing high- and low-temperature performance, but also introduces a complex multiphase system in which modifier dispersion, interfacial interactions, and phase stability are strongly dependent on material composition and processing conditions [24,25]. Achieving a suitable balance among these factors is consequently a key challenge in HDPE/CR composite-modified asphalt.

Functional additives have been introduced to improve the compatibility, dispersion, and phase stability of polymer/rubber-modified asphalt. Nano-zinc oxide (ZnO) can promote the dispersion of CR and facilitate interactions among asphalt components, thereby improving the high-temperature stability and elasticity of the modified binder [26]. Maleic anhydride (MAh), as a reactive modifier with good grafting capability, can improve the compatibility between polyethylene and asphalt and promote a more uniform distribution of the polymer phase [27,28]. Aromatic oil can increase the availability of light asphalt fractions, facilitating their absorption by CR and promoting rubber swelling. This effect can reduce CR agglomeration and segregation while improving the toughness, elasticity, and compatibility of CR with the asphalt phase [29]. However, the simultaneous incorporation of ZnO, MAh, and aromatic oil into an HDPE/CR system may produce coupled effects that cannot be readily inferred from their individual functions.

The incorporation of HDPE, CR, and multiple functional additives consequently results in a complex multiphase modification system involving polymer dispersion, rubber swelling, component redistribution, and interfacial interactions. The combined effects of these processes make the microstructural evolution and modification mechanisms more complex than those of single-modifier systems. Microstructural characterization is therefore important for clarifying the interactions among the modifiers and asphalt components and for interpreting the resulting changes in macroscopic performance. For example, Poulikakos and Sangiorgi et al. employed Fourier-transform infrared spectroscopy (FTIR) to investigate the molecular and structural characteristics of modified asphalt and the interactions between polymer modifiers and asphalt components. Their findings indicate that changes in the properties of modified asphalt are closely associated with structural and phase changes within the modified binder [30,31].

Although HDPE and CR can provide complementary effects on asphalt binder performance, previous studies have mainly focused on individual modifiers or simple HDPE/CR composite systems. The phase stability and rheological behavior of these multiphase binders depend strongly on modifier composition and processing conditions. The nano-ZnO, MAh, and aromatic oil can improve modifier dispersion, polymer–asphalt interaction, and facilitate rubber swelling, respectively. Their combined effects in HDPE/CR composite-modified asphalt remain insufficiently understood, particularly regarding modifier dispersion, phase stability, and rheological and low-temperature behavior.

In this study, an HDPE/CR composite-modified asphalt incorporating aromatic oil, nano-ZnO, and MAh was systematically investigated. An L_9_(3^3^) orthogonal design was used to optimize the processing conditions, followed by evaluation of HDPE/CR dosage combinations and auxiliary-additive effects on storage stability. DSR and BBR were employed to characterize high-temperature and low-temperature performance, while rheological changes before and after thermal storage were used as an indirect indicator of storage stability. FTIR and SEM were further used to characterize functional-group changes and modifier-phase morphology. This integrated approach clarifies the relationships among composition, processing conditions, performance, storage stability, and microstructure of the HDPE/CR modification system.

## 2. Materials

In this study, a penetration-grade 70 asphalt binder (Hunan Xinyue Asphalt Co., Ltd., Yiyang, China) and 40 mesh crumb rubber powder (Hunan Hede Rubber Co., Ltd., Yueyang, China) were selected as the matrix asphalt and rubber modifier, respectively. Other modifying additives included HDPE (CNOOC and Shell Petrochemicals Co., Ltd., Huizhou, China), nano-ZnO (Guangzhou Metallurgy (Group) Co., Ltd., Guangzhou, China), maleic anhydride (Dongguan Haobo Plastic Products Co., Ltd., Dongguan, China), and aromatic oil (Hunan Ruixida New Material Technology Co., Ltd., Yueyang, China), etc. The basic properties of the asphalt binder, including penetration, softening point, and ductility, were determined according to ASTM D5/D5M, ASTM D36/D36M, and ASTM D113 [32,33,34], respectively. The properties of matrix asphalt and other materials were provided in Table 1, Table 2, Table 3, Table 4, Table 5 and Table 6. All raw materials satisfied the corresponding technical requirements and exhibited suitable physicochemical properties for the preparation of HDPE/CR-modified asphalt binders.

All tests, including DSR and BBR, were performed in accordance with the relevant testing standards. Each group of parallel measurements was conducted in triplicate to ensure the reliability of the experimental results. The standard deviations (SD) derived from the experimental data were presented in the corresponding figures, providing a quantitative measure of the variability of the test results.

## 3. Preparation of HDPE/CR Composite-Modified Asphalt

### 3.1. Effect of Shear Conditions on Asphalt Properties

To determine the optimal shear conditions for asphalt preparation, an L_9_(3^3^) orthogonal experimental design was employed to investigate the effects of shearing speed, shearing time, and shearing temperature. Based on preliminary experiments and relevant literature [35,36], shearing speed, shearing time, and shearing temperature were selected as Factors A, B, and C, respectively, with three levels assigned to each factor (Table 7). The L_9_(3^3^) orthogonal array generated nine representative combinations, allowing the effects of the three shear parameters to be systematically evaluated with a reduced number of experiments. The dosages of HDPE, CR, and the auxiliary modifiers were determined based on preliminary laboratory investigations and relevant literature [37,38,39,40]. Throughout the experimental program, the HDPE content was fixed at 3% of the asphalt binder by mass, while the crumb rubber (CR) content was fixed at 10% of the asphalt binder by mass. Maleic anhydride (MAh) was incorporated at 1% of the asphalt binder by mass, and aromatic oil was added at 3% of the asphalt binder by mass. Nano-zinc oxide (ZnO) was added at 5% of the CR mass. These modifier dosages were kept constant across all experimental groups to eliminate the effects of compositional variations and isolate the influence of the shear parameters. Penetration, softening point, and ductility were selected as the primary response variables because they are conventional indicators of the fundamental physical properties of asphalt binders and have been widely used to assess the effects of preparation conditions on modified asphalt [39,41]. These indicators were used for preliminary optimization of the preparation process, after which the asphalt binder prepared under the optimized conditions was subjected to further rheological and performance evaluations. The orthogonal experimental design and corresponding results are presented in Table 7.

**Figure 1:**
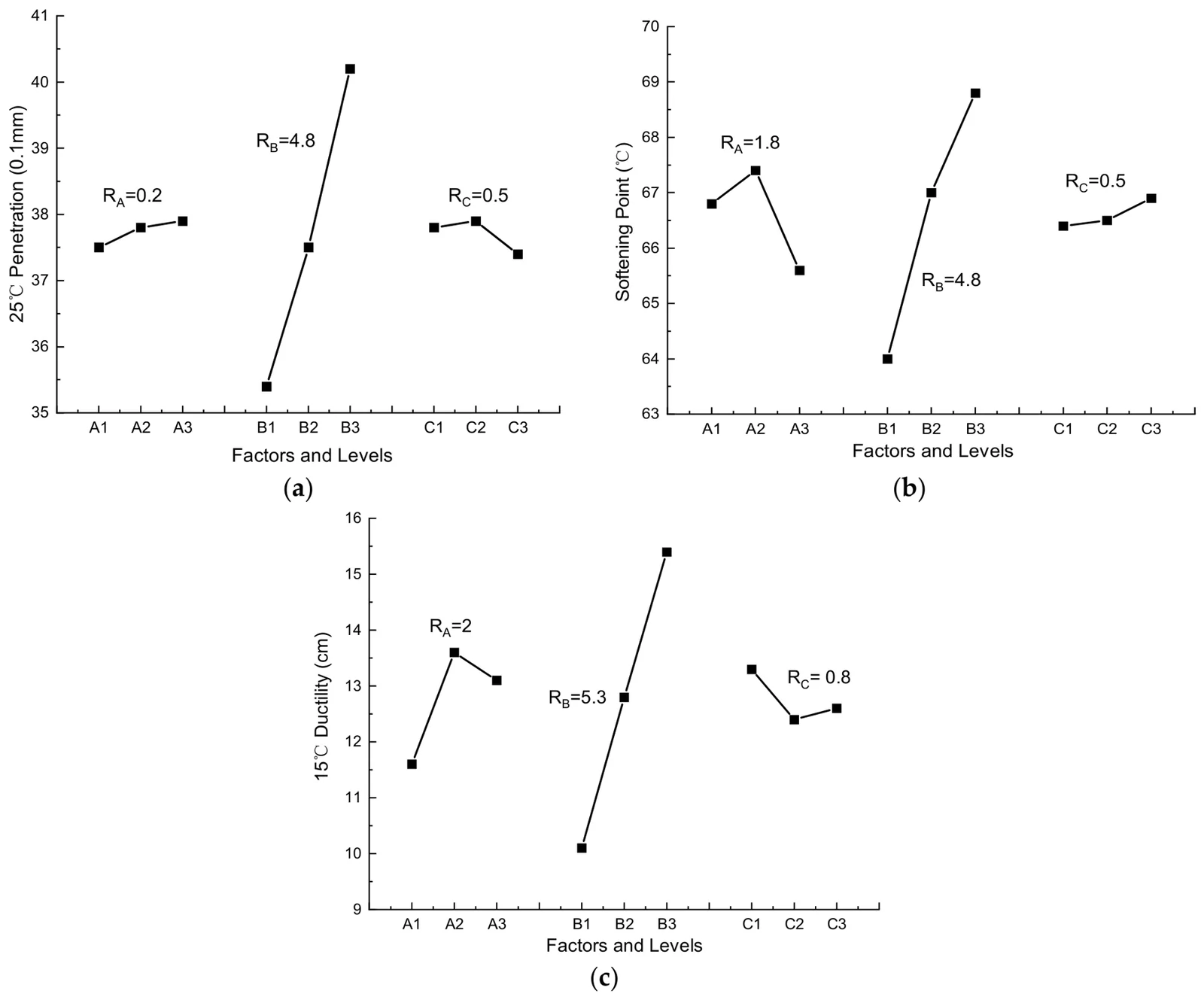
Range analysis results at different factors and levels: (**a**) penetration; (**b**) softening point; (**c**) ductility.

Range analysis was conducted to evaluate the relative effects of shear temperature, shearing time, and shearing speed on the penetration, softening point, and ductility of the composite-modified asphalt. The range value (R) represents the difference between the maximum and minimum mean responses at the three levels of each factor, with a larger R indicating a greater relative effect on the corresponding response. As shown in Figure 1, shearing time exhibited the largest R values for penetration, softening point, and ductility (4.8, 4.8, and 5.3, respectively), indicating that it was the most influential processing parameter. Shear temperature showed relatively smaller effects, with R values of 0.2, 1.8, and 2.0, respectively, whereas the corresponding R values for shearing speed were 0.5, 0.5, and 0.8. Combined with Table 7 and Figure 1, the shear temperature had a greater relative effect than shearing speed on softening point and ductility, but both were less influential than shearing time. It should be noted that range analysis reflects the relative magnitude of factor effects but does not establish their statistical significance.

The optimal preparation conditions were determined by comprehensively considering the effects of shearing speed, shearing temperature, and shearing time on penetration, softening point, and ductility. For shearing speed, an excessively high speed may accelerate asphalt aging and adversely affect its ductility and other physical properties, whereas insufficient shearing may result in inadequate dispersion of the modifiers within the asphalt matrix. Considering the balance among the effects on the evaluated binder properties, a shear speed of 6000 r/min was selected as the preferred condition. Regarding shearing time, the first experimental group exhibited relatively low values of penetration, softening point, and ductility, suggesting that the corresponding preparation conditions were not favorable for achieving the desired overall properties of the modified asphalt. Comprehensive consideration of the three performance indicators indicated that extending the shearing time to 60 min provided a better balance among the evaluated properties without causing an apparent deterioration in the fundamental properties of the asphalt binder. The effect of shear temperature on the evaluated properties was relatively limited compared with that of shearing time. Therefore, the shear temperature was selected primarily by considering the resulting penetration and softening point while minimizing potential thermal aging of the asphalt binder. Considering the need to maintain adequate ductility of the composite-modified asphalt, 180 °C was selected as the appropriate shear temperature.

Overall, considering the trade-offs among penetration, softening point, and ductility, the optimal preparation conditions were determined to be a shearing speed of 6000 r/min, a shearing time of 60 min, and a shearing temperature of 180 °C.

### 3.2. Relationship Between Modifiers Dosage and Asphalt Performance

To determine the optimal dosages of HDPE and CR, a factorial experimental design was adopted. The HDPE content was set at four levels (0%, 2%, 3%, and 4% by mass of the matrix asphalt), while the CR content was set at four levels (0%, 5%, 10%, and 15% by mass of the matrix asphalt). The combination of these two factors resulted in 16 HDPE/CR dosage combinations. For each formulation, the penetration, softening point, and ductility were determined, and Brookfield viscosity was measured at 180 °C. The obtained data were comprehensively analyzed and fitted to evaluate the effects of HDPE and CR content and to determine the optimal dosage combination for the HDPE/CR composite-modified asphalt.

**Figure 2:**
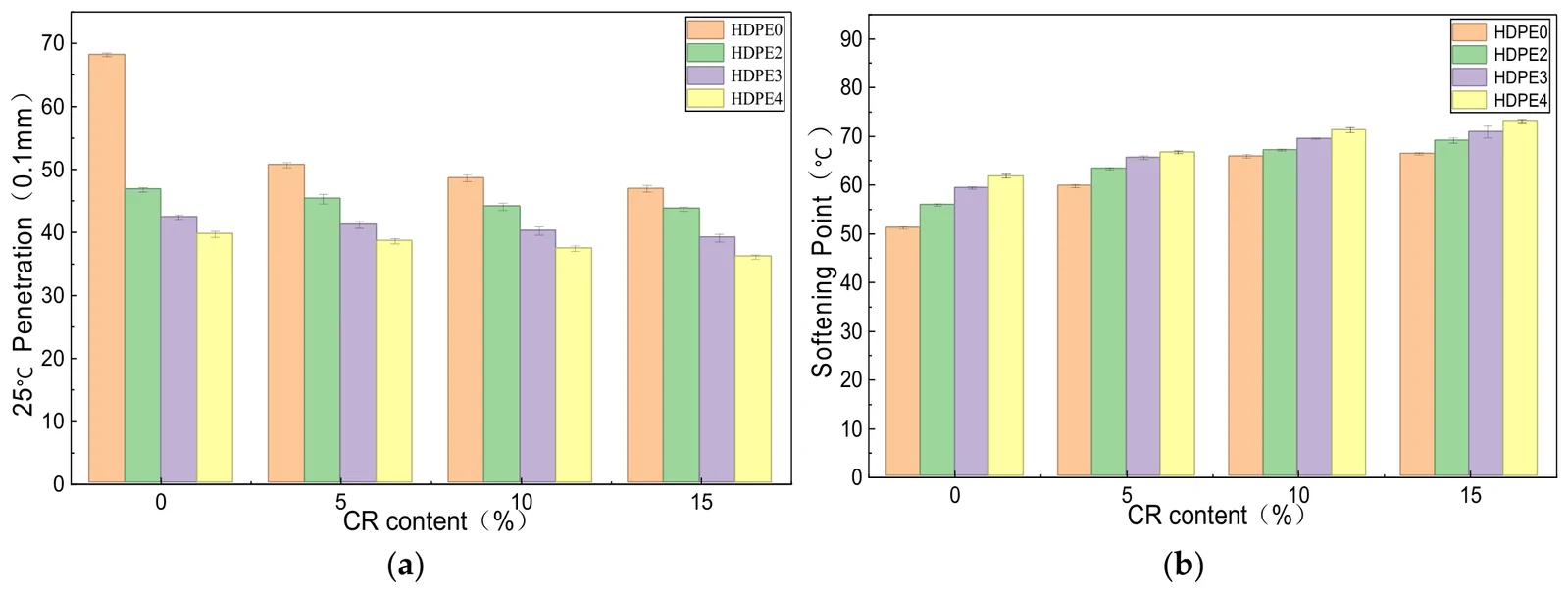

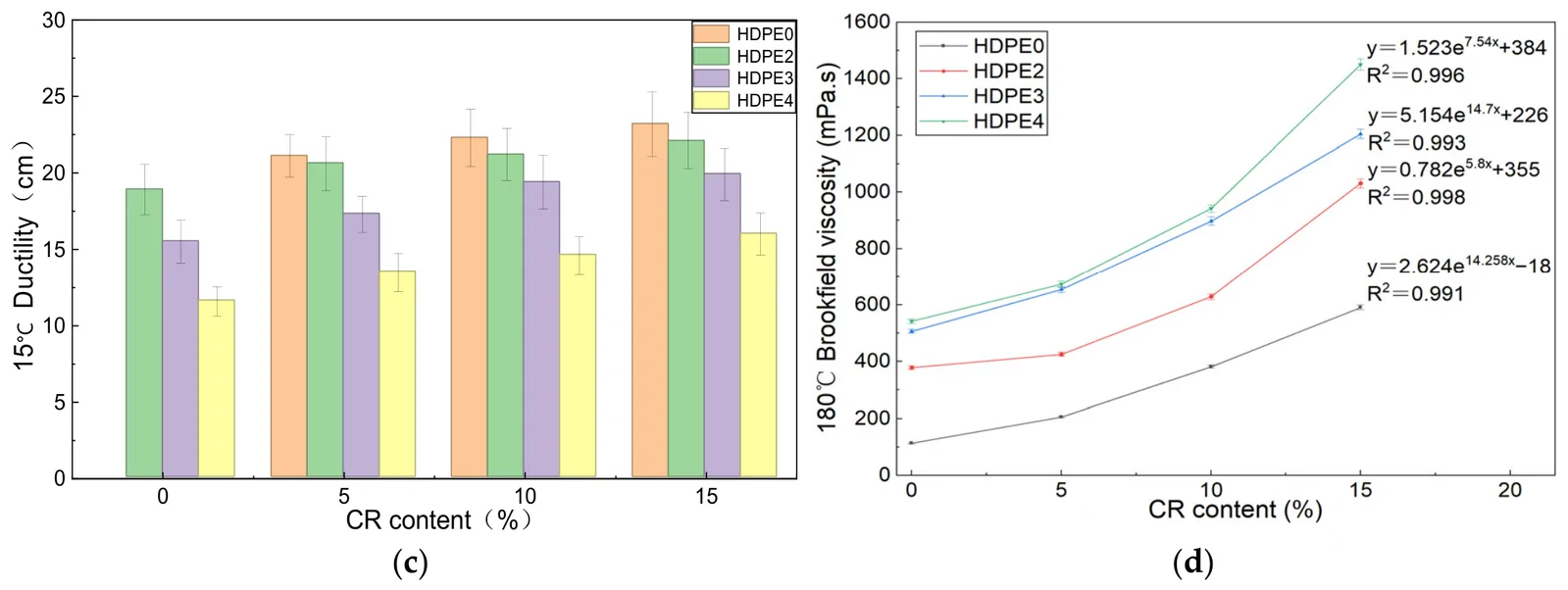
Effect of modifier dosage on different indexes: (**a**) penetration; (**b**) softening point; (**c**) ductility; (**d**) Brookfield viscosity at 180 °C (Error bars represent the standard deviation (SD)).

The effects of HDPE and crumb rubber (CR) content on the conventional properties and Brookfield viscosity of the modified asphalt are presented in Figure 2. The results demonstrate that HDPE and CR affected the asphalt in different but partially complementary ways, with HDPE exerting a more pronounced stiffening effect and CR contributing more strongly to the retention of deformation capacity. Therefore, the selection of modifier content should be considered as a balance between stiffness enhancement, high-temperature stability, low-temperature deformation capacity, and processing workability rather than on the basis of a single performance index.

As shown in Figure 2a, the penetration of the asphalt decreased continuously with increasing HDPE and CR content. At a given CR content, the reduction in penetration became more pronounced as the HDPE content increased, whereas the effect of increasing CR content was comparatively limited at a fixed HDPE content. This indicates that, within the investigated dosage range, HDPE had a stronger influence on the penetration-related stiffness of the asphalt than CR. The more pronounced effect of HDPE can be associated with the relatively rigid polymer phase introduced into the asphalt matrix, which restricts the mobility of the asphalt components and increases the resistance of the asphalt to deformation. The lower penetration observed for the composite-modified asphalt compared with the corresponding asphalts containing HDPE or CR alone further indicates that the simultaneous incorporation of the two modifiers can produce a stronger overall stiffening response. This behavior is consistent with the formation of a heterogeneous polymer/rubber-modified structure in which the dispersed modifier phases constrain the deformation of the asphalt-rich phase. However, the penetration results alone cannot establish whether the interaction between HDPE and CR is strictly synergistic, because the individual contributions of the modifiers and auxiliary additives were not independently isolated in the present experimental design.

The softening point results in Figure 2b provide further evidence of the high-temperature stiffening effect. The softening point increased with increasing content of HDPE and CR. The contribution of HDPE can reasonably be associated with the rigidity and thermal resistance of the polymer phase, which increases the resistance of the asphalt to deformation at elevated temperatures. In the case of CR, the increase in softening point is likely related not only to the presence of rubber particles but also to the absorption of lighter asphalt fractions by CR and the associated swelling of the rubber phase. Such swelling can increase the effective volume and interaction between the rubber-rich and asphalt-rich phases, thereby contributing to a more deformation-resistant internal structure. Previous studies have similarly reported improvements in the high-temperature performance of polymer- and rubber-modified asphalt; however, the magnitude of the improvement depends strongly on modifier content, processing conditions, and the degree of interaction between the modifier and asphalt phases. Thus, the increase in softening point should be interpreted as the combined result of modifier-induced stiffening and changes in the internal phase structure rather than as evidence of a single modification mechanism.

In contrast to penetration and softening point, ductility at 15 °C exhibited an opposing response to the two modifiers, as shown in Figure 2c. Increasing HDPE content gradually reduced ductility, indicating that the increase in asphalt stiffness associated with the polymer phase was accompanied by a reduction in the capacity for tensile deformation at low temperature. Conversely, increasing CR content increased ductility, particularly when the CR content reached 15%, at which point the ductility exceeded 20 cm. This difference highlights the complementary roles of the two modifiers: HDPE primarily contributes to stiffness and high-temperature deformation resistance, whereas CR provides a greater contribution to flexibility and deformation accommodation. The limited improvement in ductility at relatively low CR content may be related to the insufficient volume fraction of the rubber phase to exert a substantial elastic effect. In addition, the swelling of CR depends on the absorption of compatible light components from the asphalt, meaning that the effectiveness of CR in restoring deformation capacity is also affected by the composition of the surrounding asphalt phase. Therefore, increasing CR content can partially compensate for the loss of ductility associated with HDPE modification, but excessive modifier content may simultaneously increase asphalt viscosity and processing difficulty.

The Brookfield viscosity results at 180 °C further demonstrate the trade-off introduced by increasing modifier content. As shown in Figure 2d, viscosity increased continuously with increasing CR content for all investigated HDPE contents, and the exponential fitting equations exhibited high coefficients of determination (R^2^ > 0.99). At the same CR content, asphalts containing higher HDPE content exhibited higher viscosity, indicating that HDPE made a more pronounced contribution to viscosity enhancement within the investigated dosage range. The increase in viscosity can be attributed to the combined effects of the rigid polymer phase, CR swelling, and interactions between the modifiers and the asphalt components. In particular, swelling of CR increases the effective volume of the rubber phase and restricts the flow of the surrounding asphalt, while the polymer phase further limits molecular mobility. Consequently, the viscosity increase should be regarded as a rheological consequence of the formation and development of the modifier-rich structure rather than simply as an isolated increase in flow resistance [42,43].

From an engineering perspective, an increase in viscosity may be beneficial because it indicates greater resistance to flow and is generally associated with improved high-temperature stability. However, excessively high viscosity can negatively affect mixing, pumping, coating, and construction operations. The simultaneous increase in stiffness and viscosity with increasing HDPE and CR content therefore imposes a practical upper limit on modifier dosage. In contrast, the increase in ductility with CR provides a means of offsetting the loss of deformation capacity associated with HDPE. The results consequently indicate that HDPE and CR play partially complementary roles in the composite modification system: HDPE contributes predominantly to stiffness, softening-point improvement, and viscosity enhancement, whereas CR contributes more strongly to ductility while also increasing stiffness-related and viscous responses.

Considering the combined effects of penetration, softening point, ductility, and Brookfield viscosity, the formulation containing 3% HDPE and 15% CR was selected as the preferred composition within the investigated dosage range. Importantly, this selection should be understood as an optimization within the tested dosage window rather than as evidence that 3% HDPE and 15% CR represent universal optimum content. The selected formulation provides a comparatively balanced combination of stiffness, high-temperature deformation resistance, low-temperature deformation capacity, and processing workability and was therefore adopted for the subsequent rheological and compatibility evaluations.

### 3.3. Compatibility Evaluation

Aromatic oil, nano zinc oxide, and maleic anhydride were selected to be added to the modified asphalt, and the results were compared and observed. The dosage of compatible stabilizers is determined according to relevant references and existing experimental studies, and the dosage scheme is shown in Table 8.

The MSCR parameters have been widely used to characterize the permanent deformation resistance of polymer-modified asphalt binders and show a strong correlation with rutting performance [44,45]. Furthermore, previous studies have demonstrated that differences in *Jnr* measured from different portions of thermally conditioned binders can reflect modifier migration and rheological heterogeneity resulting from storage separation [46,47]. Therefore, the variation in *Jnr* between the upper section and the bottom section after thermal storage was used as an indirect rheological indicator of storage stability to evaluate the compatibility. The separation index (*SI*) was calculated by Equation (1) [48]. The MSCR tests were conducted at 64 °C, with a loading period of 1 s followed by an unloading and recovery period of 9 s for each creep cycle. After 48 h of storage at 163 °C, samples from the upper one-third section and bottom one-third section of the aluminum tube were examined. The test results are shown in Table 9. The CRM in the program is CR-modified asphalt.

(1) $${SI}_{\sigma }=\frac{{\left(Jnr\right)}_{max}-{\left(Jnr\right)}_{avg}}{{\left(Jnr\right)}_{avg}}\times 100\%$$

where ${SI}_{\sigma }$ is the separation index at σ = 0.1 or 3.2 kPa; *(Jnr)max* and *(Jnr)avg* are the maximum *Jnr* value and average value of the upper and bottom sections in the MSCR test.

As shown in Table 9, the investigated formulations differed markedly in their rheological separation behavior. The PC and CRM binders exhibited substantially higher separation indices than the other modified binders. In particular, CRM showed the highest *SI* values, reaching 41.2% and 33.0% at 0.1 and 3.2 kPa, respectively. The pronounced differences in *Jnr* between the upper section and the bottom section indicate significant rheological heterogeneity after thermal storage, suggesting relatively poor storage stability. For the HDPE/CR system without auxiliary additives, the pronounced difference in *Jnr* between the upper section and the bottom section may be associated with component redistribution during prolonged heating, in agreement with previous observations of storage-induced rheological differences in crumb-rubber-modified asphalt binders [48].

The addition of auxiliary additives reduced the *SI* values to different extents. The *SI* values of F3N5 were reduced to 24.9% and 21.4%, while those of M1 exhibited lower values of 16.4% and 8.2% at 0.1 and 3.2 kPa, respectively. The M1N5F3 formulation exhibited the lowest *SI* values, 6.9% and 7.7%, corresponding to reductions of approximately 77% and 72% relative to PC. The smaller differences in *Jnr* between the upper section and the bottom section indicate a more uniform rheological distribution after thermal storage. The reduction in *SI* demonstrated that the auxiliary additives contributed to improving the storage stability of the composite-modified binder. However, the present results do not allow the individual contribution of maleic anhydride, nano-ZnO, and aromatic oil to be separated.

The progressively lower *SI* values from PC to F3N5, M1, and M1N5F3 indicate that the incorporation of auxiliary additives was associated with improved rheological uniformity after thermal storage. A possible explanation is that these additives altered modifier dispersion and/or interactions among the constituent phases. Nevertheless, this interpretation remains inferential because the present rheological results do not provide direct evidence of specific molecular interactions or chemical reactions. Therefore, the observed improvement should be interpreted primarily in terms of enhanced phase-distribution stability rather than as evidence of a confirmed molecular-level compatibility mechanism. Among the investigated formulations, M1N5F3 exhibited the lowest *SI* values and thus the most uniform rheological distribution after thermal storage, supporting its selection as the preferred auxiliary-additive combination within the investigated experimental range.

### 3.4. Preparation of Modified Asphalt

**Figure 3:**
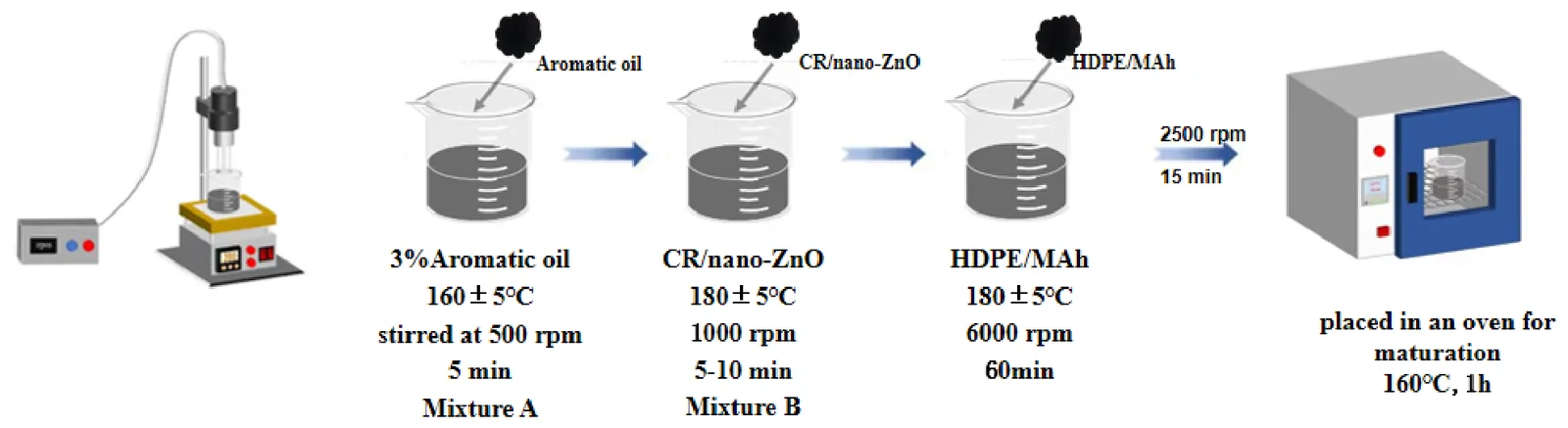
Flow chart of modified asphalt preparation.

Based on the presented results and considering the feasibility and stability of the preparation process, the mixing temperature, shearing speed, and mixing duration were determined. The selected formulation contained 15% CR and 3% HDPE, while nano-ZnO was added at 5% of the mass of CR. Aromatic oil and maleic anhydride were added at 3% and 1% of the mass of the matrix asphalt, respectively. The specific preparation process was shown in Figure 3.

1. Aromatic oil (3% by mass of the matrix asphalt) was added to the matrix asphalt. The mixture was heated to 160 °C and stirred at 500 rpm for 5 min to obtain a relatively homogeneous Mixture A.
2. CR (15%, by mass of the matrix asphalt) and nano-ZnO (5%, by mass of CR) were gradually introduced after Mixture A was heated to 180 °C. During this process, the mixture was slowly sheared at 1000 rpm for 5–10 min to ensure uniform dispersion of the CR and nano-ZnO, forming Mixture B.
3. HDPE (3%, by mass of the matrix asphalt) and MAh (1%, by mass of the matrix asphalt) were gradually introduced into Mixture B. The mixture was then subjected to high-speed shearing at 6000 rpm for 60 min while maintaining the temperature at 180 °C to ensure sufficient dispersion and incorporation of the modifiers.
4. Following high-speed shearing, the mixture was subjected to low-speed shearing at 2500 rpm for no more than 15 min to improve its homogeneity and minimize excessive air entrainment. The resulting HDPE/CR composite-modified asphalt was then transferred to an oven maintained at 160 °C for 1 h. During this period, the binder was gently stirred at 10-min intervals to facilitate the removal of entrapped air bubbles.

## 4. Performance of HDPE/CR Composite-Modified Asphalt

### 4.1. High-Temperature Rheological Properties

**Figure 4:**
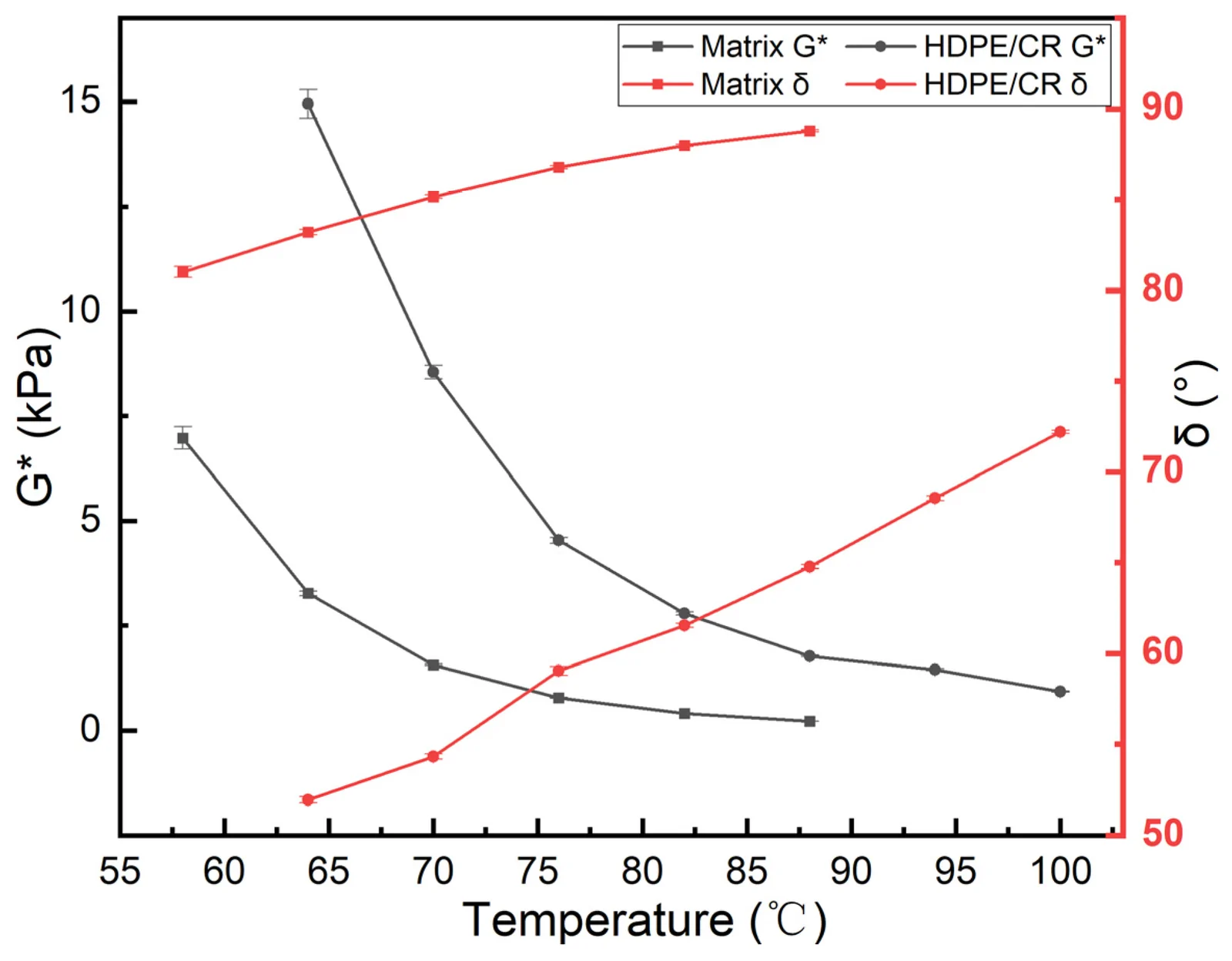
DSR scan results with the temperature of different asphalts. (Error bars represent the standard deviation (SD)).

To characterize the high-temperature rheological behavior of the HDPE/CR composite-modified asphalt, a temperature sweep test was conducted using a dynamic shear rheometer (DSR) on the matrix asphalt and HDPE/CR composite-modified asphalt. During the test, the complex modulus (G*) and phase angle (δ) were measured as functions of temperature. The complex modulus G*, which represents the overall resistance of the asphalt binder to deformation under oscillatory loading, was used to characterize the stiffness of the binders, whereas δ, which represents the phase lag between shear stress and shear strain, was used to characterize the relative contributions of viscous and elastic responses. The high-temperature performance parameter G^∗^/sinδ was subsequently calculated from the measured G* and phase angle δ. According to the Superpave criterion, the temperature corresponding to G*/sinδ = 1.0 kPa for the original binder was determined by linear interpolation between the adjacent experimental temperature points and was defined as the high-temperature failure temperature. The temperature sweep was performed over the range shown in Figure 4.

**Figure 5:**
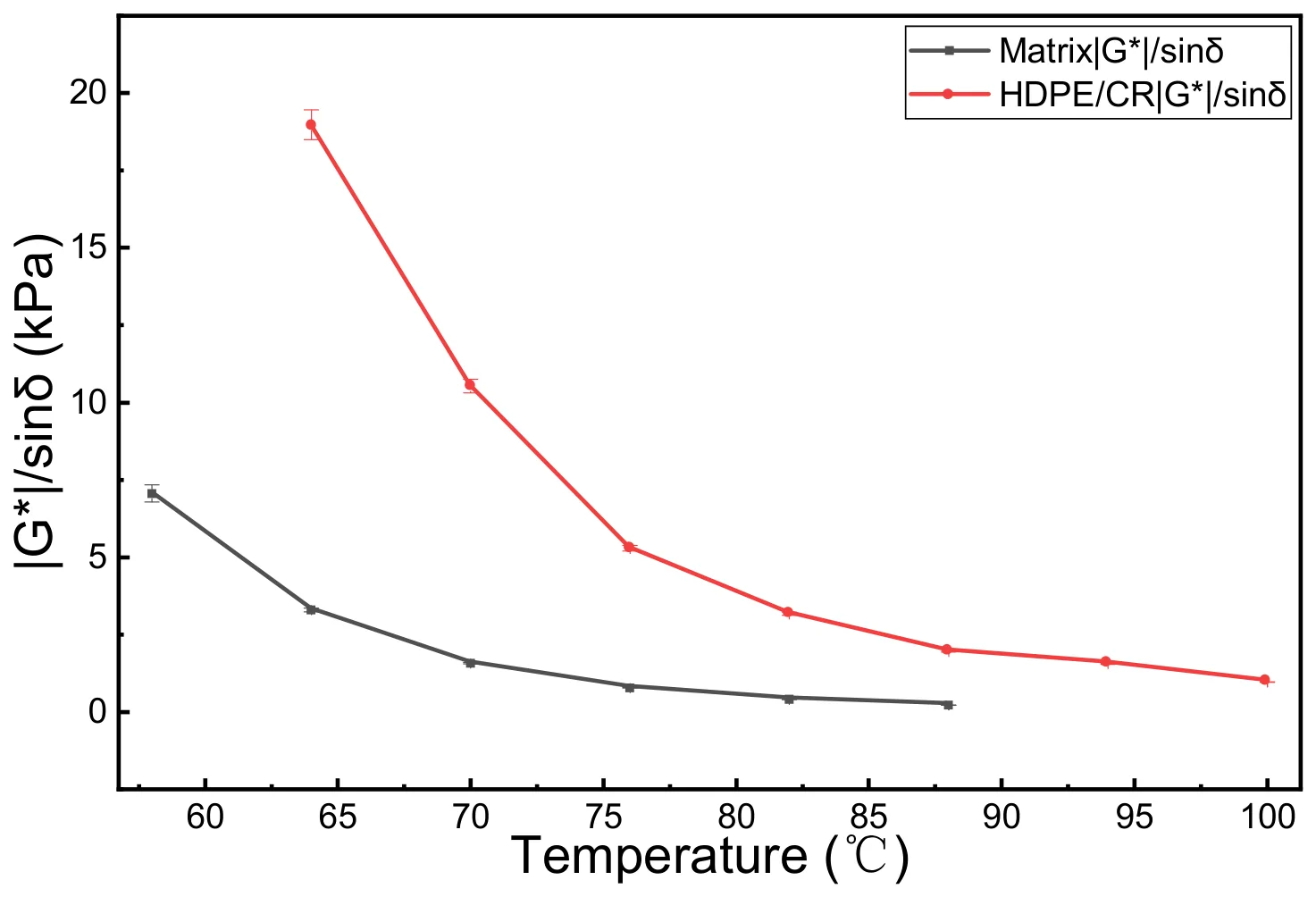
G*/sinδ scan results with the temperature of different asphalts. (Error bars represent the standard deviation (SD)).

As shown in Figure 4 and Figure 5, the complex modulus (G*), phase angle (δ), and rutting factor (G*/sinδ) were used to evaluate the high-temperature rheological properties of the matrix asphalt and HDPE/CR composite-modified asphalt. Compared with the matrix asphalt, the HDPE/CR composite-modified asphalt exhibited higher G^∗^ values over the entire test temperature range, indicating increased binder stiffness and enhanced resistance to high-temperature deformation. For both binders, the phase angle (δ) increased with increasing temperature, indicating an increased viscous contribution in the viscoelastic response. However, the HDPE/CR composite-modified asphalt showed lower δ values than the matrix asphalt, suggesting an increased elastic response after modification. This behavior may be associated with the formation of a more stable modified binder structure and reduced mobility of asphalt components resulting from the incorporation of HDPE and CR. In addition, the G*/sinδ values of the HDPE/CR composite-modified asphalt were consistently higher than those of the matrix asphalt, indicating improved resistance to permanent deformation. According to the Superpave criterion, a higher G*/sinδ value represents enhanced high-temperature rutting resistance of asphalt binders. Therefore, the increased G*/sinδ values demonstrate that the combined effects of HDPE, CR, and auxiliary additives effectively enhanced the high-temperature rheological performance and rutting resistance of the asphalt binder.

### 4.2. Low-Temperature Crack Resistance

**Figure 6:**
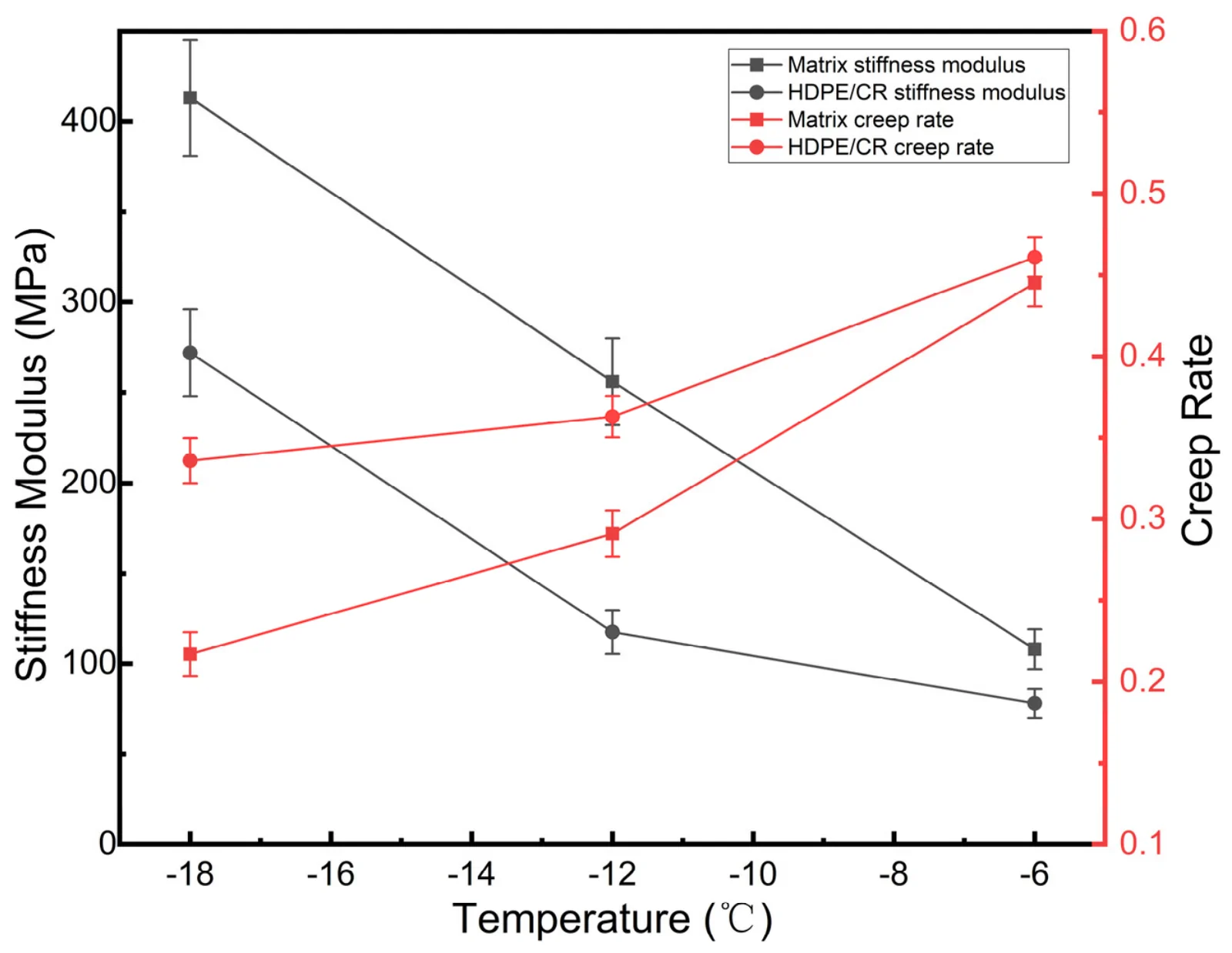
BBR scan results with the temperature of different asphalts. (Error bars represent the standard deviation (SD)).

To characterize the low-temperature cracking resistance of the HDPE/CR composite-modified asphalt, a bending beam rheometer (BBR) test was conducted after short-term aging by the rolling thin-film oven test (RTFOT) and long-term aging by a pressurized aging vessel (PAV) of the matrix asphalt and HDPE/CR composite-modified asphalt. The stiffness modulus (S) and creep rate (m)-value at 60 s, denoted as S (60) and m (60), respectively, were determined at −6, −12, and −18 °C in accordance with AASHTO T 313 [49]. The variations in S (60) and m (60) with temperature were presented in Figure 6.

As shown in Figure 6, the stiffness modulus of both binders increased as the temperature decreased, indicating an increase in resistance to creep deformation under low-temperature conditions. However, the HDPE/CR composite-modified asphalt exhibited a substantially lower stiffness modulus than the matrix asphalt at the corresponding temperatures. In particular, the stiffness modulus of the composite-modified asphalt increased relatively gradually from −6 to −12 °C, indicating that the modified binder retained greater low-temperature compliance than the matrix asphalt. This behavior suggests that the combined modification system reduced the low-temperature stiffness of the asphalt binder and may therefore be beneficial for mitigating thermally induced cracking.

The (m)-value of both binders decreased with decreasing temperature, indicating a reduced creep rate and, consequently, a diminished ability to accommodate thermally induced stresses under increasingly severe low-temperature conditions. Compared with the matrix asphalt, the HDPE/CR composite-modified asphalt exhibited a relatively slight decrease in the (m)-value. At −18 °C, the (m)-value of the HDPE/CR composite-modified asphalt remained at 0.336, whereas that of the matrix asphalt decreased to below 0.30. The relatively higher (m)-value, together with the lower stiffness modulus, indicates that the composite-modified asphalt possessed a greater ability to accommodate thermal contraction and relieve thermally induced stresses at low temperatures. Overall, the BBR results demonstrated that the HDPE/CR composite modification system improved the low-temperature rheological response of the asphalt binder, as evidenced by its lower stiffness modulus and relatively higher creep rate. However, because the present experimental design evaluated the combined modification system rather than the individual effects of HDPE, CR, aromatic oil, nano-ZnO, and maleic anhydride separately, the observed improvement cannot be attributed to any single modifier or compatibilizer alone.

## 5. Modification Mechanism

### 5.1. FTIR

Infrared spectra of HDPE, matrix asphalt and HDPE/CR samples were measured by a Nicolet iS10 Fourier Transform Infrared Spectrometer. The changes in the functional groups of asphalt after modification were analyzed to reveal the mechanism of modification of matrix asphalt by modifiers.

**Figure 7:**
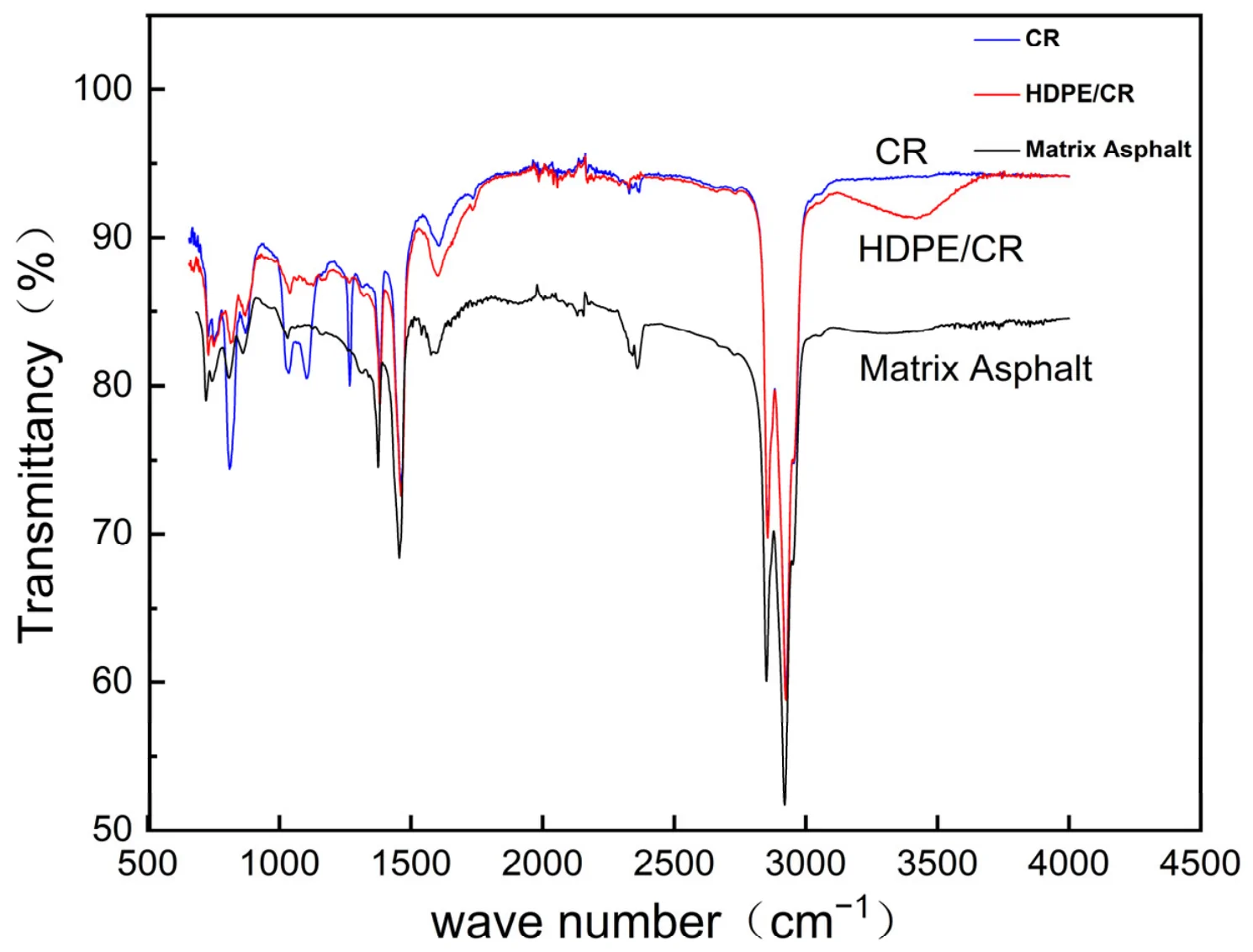
Comparison of infrared spectra of different asphalts.

As shown in Figure 7, the FTIR spectrum of CR exhibits five distinct absorption peaks in the functional group region from 1300 to 600 cm^−1^, whereas fewer distinct peaks are observed in the spectrum of the HDPE/CR composite-modified asphalt. This difference may be associated with the overlapping or masking of characteristic absorption bands from the different modifier components in the composite system. Such spectral changes are commonly observed in complex polymer-modified asphalt systems and can reflect changes in the chemical environment and interactions among the constituent components; however, they should not be interpreted as direct evidence of specific chemical reactions without complementary characterization [50].

In the 1300–600 cm^−1^ region, the absorption intensities at 1097.60, 1029.21, and 804.42 cm^−1^ were reduced in the HDPE/CR composite-modified asphalt compared with those of CR. These changes suggest that the molecular environments associated with the corresponding functional groups were altered after the incorporation of HDPE and the other components of the composite modification system. The disappearance of the absorption bands observed at 2325.09 and 2327.62 cm^−1^ in the CR spectrum may also indicate changes in the corresponding molecular vibrations or interactions among the components. However, the disappearance or attenuation of individual FTIR bands alone cannot conclusively demonstrate the rupture of a specific C≡C bond or the occurrence of a chemical reaction.

The absorption features around 804.42 cm^−1^ should likewise be interpreted cautiously. Although changes in this region may be associated with variations in the molecular structure or vibrational environment of the HDPE/CR composite system, the present FTIR results do not provide sufficient evidence to confirm C–H bond opening, chemical cross-linking, or a specific change in the three-dimensional structure of HDPE. Such reaction pathways would require complementary evidence from techniques capable of directly characterizing chemical bonding or molecular structure. Therefore, the FTIR results in Figure 7 primarily indicate that the incorporation of HDPE and CR altered the spectral characteristics of the asphalt binder and suggest the presence of interactions among the components. Overall, the observed spectral changes are consistent with the formation of a modified asphalt system involving interactions between the asphalt binder and the modifiers; however, whether these changes arise exclusively from physical blending or also involve specific chemical reactions cannot be conclusively determined from FTIR analysis alone [11].

### 5.2. SEM

The surface morphologies of matrix asphalt, CRMA, and HDPE/CRMA were observed by scanning electron microscopy. It can be used to observe the distribution of different modifiers in asphalt.

**Figure 8:**
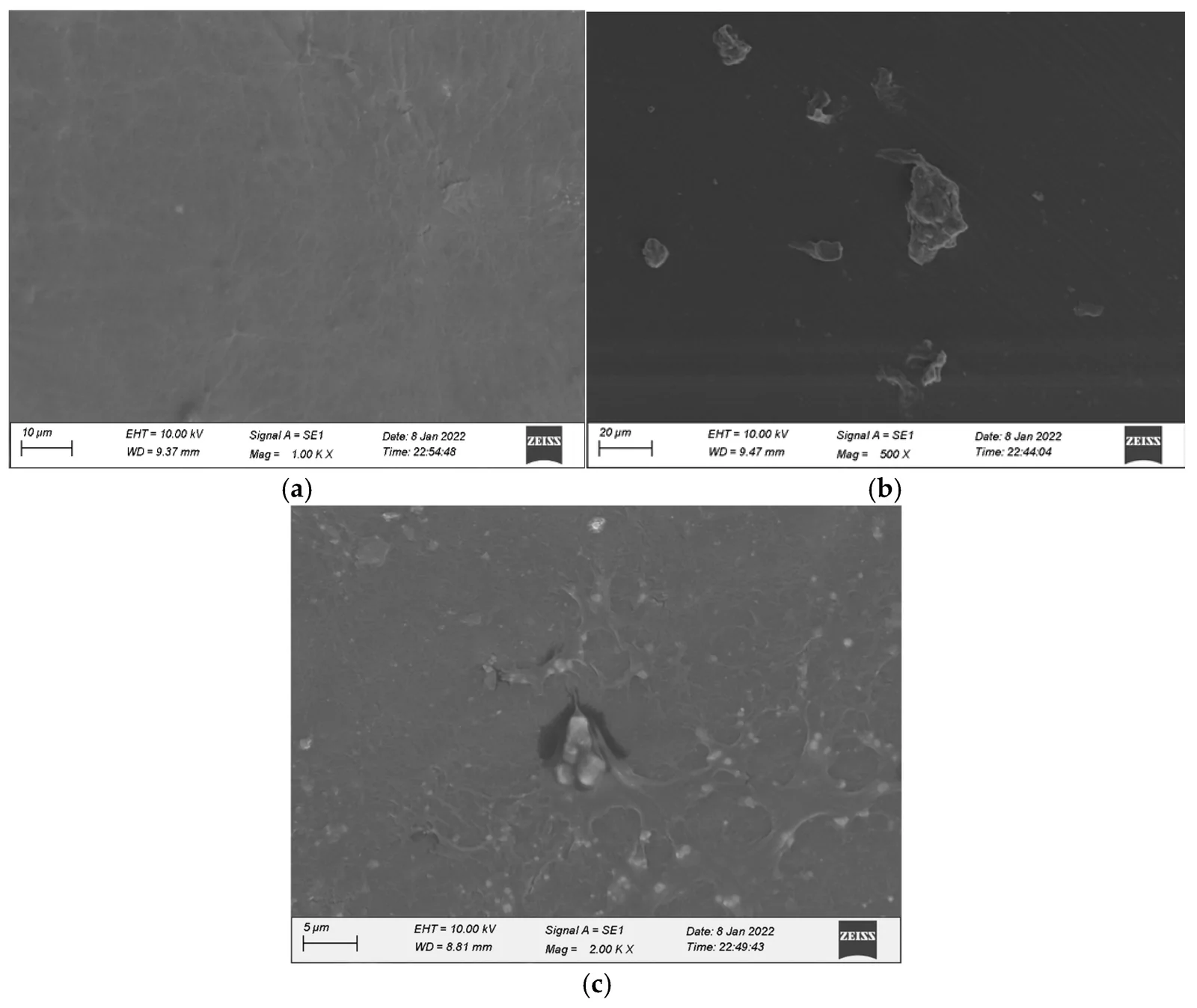
Scanning electron microscopy results of asphalts: (**a**) matrix asphalt; (**b**) CRMA; (**c**) HDPE/CRMA.

As shown in Figure 8a, the surface of the matrix asphalt exhibited a relatively flat and homogeneous morphology, with no obvious large-scale aggregates or irregular structures observed in the grey and black regions. This indicates that the matrix asphalt had a relatively uniform surface morphology before modification. In Figure 8b, several irregular flocculent white features with characteristic dimensions of approximately 10–20 μm can be observed on the asphalt surface. These features may be associated with rubber powder particles or rubber-rich domains. During the shear preparation process, the rubber particles may have interacted with and absorbed part of the light components of the asphalt, resulting in changes in their morphology and interfacial characteristics. The irregular morphology and locally aggregated features may also be related to the swelling of rubber particles and their interaction with the asphalt matrix under the combined effects of temperature and shear [51].

As shown in Figure 8c, white globular and flocculent features, together with relatively continuous white film-like regions, can be observed on the surface of the HDPE/CR composite-modified asphalt. Compared with the matrix asphalt and the rubber-modified asphalt, the composite-modified asphalt exhibited a more heterogeneous multiphase morphology, suggesting that the incorporation of HDPE further altered the phase distribution and surface microstructure of the binder. The observed globular and flocculent features may correspond to modifier-rich domains, while the film-like regions may be associated with the interfacial distribution of the polymer/rubber-rich phase. However, SEM provides primarily morphological information and cannot directly confirm the molecular weight of HDPE, its dissolution state, or specific molecular-level interactions with the asphalt matrix. Therefore, the observed morphology should be interpreted as evidence of changes in phase distribution and dispersion rather than direct evidence that HDPE was incompletely dissolved in the asphalt.

It should also be noted that the SEM observations in Figure 8 are primarily qualitative. Although differences in particle morphology, aggregation, and phase distribution can be visually identified, quantitative parameters such as particle-size distribution, phase-area fraction, dispersion uniformity, or interparticle distance were not obtained in the present analysis. Quantitative image analysis could provide more objective evidence for evaluating the dispersion state and microstructural homogeneity of the modifiers. Therefore, the SEM results in this study are mainly used to provide qualitative morphological evidence supporting the formation of a heterogeneous composite-modified asphalt system, rather than to establish a specific molecular-level modification mechanism.

## 6. Conclusions

In this study, an HDPE/crumb rubber (CR) composite-modified asphalt binder was developed, and the effects of preparation conditions, modifier interactions, and structural evolution on binder performance were systematically investigated. The main conclusions are summarized as follows:

1. The combination of HDPE and CR provides an effective approach for balancing stiffness enhancement and flexibility improvement in asphalt binders. Under the investigated preparation conditions, the preferred preparation process promoted the dispersion and interaction of the polymer and rubber phases, resulting in a composite-modified binder with improved overall performance compared with the matrix asphalt. The identified preparation conditions should therefore be regarded as preferred conditions within the investigated experimental range rather than as statistically validated global optimum conditions.
2. Within the investigated modifier dosage range, the selected HDPE/CR composition and auxiliary-additive combination provided a favorable balance of conventional, rheological, and low-temperature properties and demonstrated the potential for value-added utilization of waste tire rubber and HDPE in asphalt binders. However, the selected composition and preparation conditions represent a preferred combination under the specific experimental conditions and dosage ranges investigated in this study. In addition, the individual contributions and interaction effects of the auxiliary additives were not completely separated. Future studies should therefore investigate the independent and synergistic effects of HDPE, CR, and auxiliary additives using more comprehensive experimental designs, replicated statistical analysis, and long-term durability evaluations.
3. Rheological evaluation demonstrated that the HDPE/CR composite-modified asphalt exhibited enhanced resistance to high-temperature deformation while maintaining improved low-temperature performance relative to the base asphalt binder. The changes in rheological parameters before and after thermal storage further indicated relatively stable modifier distribution within the composite system. However, the MSCR results were used as an indirect rheological indicator of storage stability rather than as a direct measurement of compatibility.
4. The modification mechanism of the HDPE/CR composite system involved the combined effects of polymer and rubber incorporation, modifier dispersion, phase distribution, and modifier–asphalt interfacial interactions. FTIR and SEM results indicated changes in the chemical-group environment and heterogeneous multiphase morphology, suggesting altered interactions and spatial distribution among the constituent phases. The auxiliary additives may have further promoted modifier dispersion and interfacial interactions, contributing to a relatively stable composite structure. However, these results do not provide sufficient evidence to establish specific chemical reactions or molecular-level pathways; the modification mechanism is therefore considered to involve physical blending, phase redistribution, and possible interfacial interactions.

## Figures and Tables

**Table 1:** Basic performance indexes of 70# grade matrix asphalt.

Test Items		Test Results	Technical Requirements
Penetration (0.1 mm)		68.7	60–80
Penetration index		0.14	−1.5~+1.1
Softening point (°C)		50.7	≥46
15 °C ductility (cm)		121	≥100
10 °C ductility (cm)		34	≥15
60 °C dynamic viscosity (Pa∙s)		269	≥180
Flash point (°C)		289	≥260
Relative density		1.04	-
Wax content (%)		1.0	≤2.2
Solubility (%)		99.7	≥99.5
Residue after RTFOT	Mass loss (%)	0.13	−0.8~+0.8
	Penetration ratio (%)	68.4	≥61
	10 °C ductility (cm)	13.8	≥6
	15 °C ductility (cm)	74.5	-

**Table 2:** Basic performance indexes of crumb rubber.

Test Items	Test Results	Technical Requirements
Sieve residue (%)	7.5	<10
Relative density	1.23	1.10~1.30
Moisture content (%)	0.5	<1.0
Metal content (%)	0.05	<0.05
Fiber content (%)	0.03	<1.0
Ash (%)	8	≤8
Natural rubber content (%)	38	≥30
Acetone extract (%)	10	≤22
Carbon black content (%)	34	≥28
Rubber hydrocarbon content (%)	58	≥48

**Table 3:** Basic performance indexes of HDPE.

Density (g/cm^3^)	Tensile Strength (MPa)	Melting Point (°C)	Crystallinity (%)	Breaking Elongation (%)	Catalytic Temperature (°C)
0.954	16.7	140	87	650	100~70

**Table 4:** Basic performance indexes of nano zinc oxide.

Test Items	Test Results	Technical Requirements
Exterior condition	White	White powder
ZnO content (%)	99.9	≥99.8
Loss of dehumidification (%)	0.3	≤0.5
Loss of ignition (%)	1.2	≤3
Average particle size (nm)	19	≤20

**Table 5:** Basic performance indexes of maleic anhydride.

Test Items	Test Results	Technical Requirements
Color	White	White
Melting point (°C)	53	52~55
Water solubility (g/mL)	79	70~80
Refractive index	1.54	1.5~1.6
Density (g/cm^3^)	1.14	1.10~1.21

**Table 6:** Basic performance indexes of aromatic oils.

Test Items	Test Results	Technical Requirements
Color	Dark brown, brown	Brown
Flashpoint (°C)	247	≥200
100 °C Dynamic viscosity (Pa∙s)	44	35~45
Aromatic hydrocarbon content (%)	85	≥80
Density (g/cm^3^)	1.06	1.04~1.06
Volatile component	None	None

**Table 7:** Orthogonal test scheme.

Test Number	Factors and Levels			Evaluation Indexes		
	Shearing Speed (A) (r/min)	Shearing Time (B) (min)	Shearing Temperature (C) (°C)	Penetration (0.1 mm)	Softening Point (°C)	15 °C Ductility (cm)
1	5000	30	170	35.1	63.6	9.3
2	5000	45	180	37.6	67.6	11.1
3	5000	60	190	39.8	69.2	14.4
4	6000	30	180	35.8	64.7	10.9
5	6000	45	190	37.1	67.8	13.3
6	6000	60	170	40.5	69.8	16.5
7	7000	30	190	35.4	63.7	10.1
8	7000	45	170	37.9	65.7	13.9
9	7000	60	180	40.4	67.4	15.3

**Table 8:** Compatibility program.

Test Group	Dosage Scheme
F3N5	3% HDPE + 15% CR + 3% aromatic oil + 5% nano zinc oxide
M1	3% HDPE + 15% CR + 1% maleic anhydride
M1N5F3	3% HDPE + 15% CR + 1% maleic anhydride + 5% nano zinc oxide + 3% aromatic oil
PC	3% HDPE + 15% CR

**Table 9:** Separation test results of composite-modified asphalt with different compatibility schemes at 64 °C.

Test Group	*Jnr*, 0.1 (kPa^−1^)			*Jnr*, 3.2 (kPa^−1^)		
	Upper Sample	Bottom Sample	*SI*_0.1_	Upper Sample	Bottom Sample	*SI*_3.2_
F3N5	0.926	0.557	24.9	1.326	0.858	21.4
M1	1.074	1.496	16.4	1.454	1.713	8.2
M1N5F3	0.596	0.684	6.9	0.766	0.894	7.7
PC	2.563	4.736	29.8	2.738	4.817	27.5
CRM	1.023	2.454	41.2	1.376	2.732	33.0

## Data Availability

The original contributions presented in this study are included in the article. Further inquiries can be directed to the corresponding author.
